# Is Wheat Glutenin Extract Intrinsically Allergenic? Evaluation Using a Novel Adjuvant-Free Mouse Model of Systemic Anaphylaxis

**DOI:** 10.3390/ijms242417247

**Published:** 2023-12-08

**Authors:** Rick Jorgensen, Haoran Gao, Tamil Selvan Arul Arasan, Chris Van Antwerp, Vaisheswini Sundar, Perry K. W. Ng, Venu Gangur

**Affiliations:** 1 Food Allergy and Immunology Laboratory, Department of Food Science and Human Nutrition, Michigan State University, East Lansing, MI 48823, USA; jorgen70@msu.edu (R.J.); gaohaora@msu.edu (H.G.); arultami@msu.edu (T.S.A.A.); vanant29@msu.edu (C.V.A.); sundarva@msu.edu (V.S.); 2 Cereal Science Laboratory, Department of Food Science and Human Nutrition, Michigan State University, East Lansing, MI 48823, USA; ngp@msu.edu

**Keywords:** glutenin allergy, skin sensitization, systemic anaphylaxis, IgE, mouse model

## Abstract

Wheat is a prominent allergenic food that can trigger life-threatening anaphylaxis. Presently, it remains unclear whether wheat glutenin (WG) extract possesses inherent sensitization potential independently, without the use of adjuvants, and whether it can sensitize mice to the extent of inducing life-threatening systemic anaphylaxis. In this study, we tested the hypothesis that repeated skin exposures to WG extract without adjuvant will sensitize mice with the resultant anaphylactic reaction upon systemic WG challenge. Balb/c mice were bred and maintained on a strict plant protein-free diet and were repeatedly exposed to a WG extract or vehicle once a week for 9 weeks. WG-specific (s)IgE and total (t)IgE levels were quantified. Mice were challenged with WG extract to induce anaphylactic reactions as measured by hypothermic shock response (HSR) and mucosal mast cell degranulation response (MMCR). We also conducted proteomic analysis of 120 spleen immune markers. These skin-sensitized mice exhibited exposure-dependent IgE responses and near-fatal anaphylaxis upon challenge. Proteomic analysis identified seven dramatically elevated immune biomarkers in anaphylactic mice. These data reveal that WG is intrinsically allergenic, and that chronic skin exposure to WG extract can prime the mice for potentially fatal anaphylaxis.

## 1. Introduction

Wheat hypersensitivity is estimated to affect 0.4–3% in the United States [1,2,3]. Symptoms of wheat allergy can manifest as urticaria/angioedema, asthma, allergic rhinitis, abdominal pain, vomiting, acute dermatitis, anaphylaxis, and WDEIA [4,5,6,7]. There is currently no cure for wheat allergy [8]. Affected individuals must maintain a gluten-free/wheat-free diet as the primary method of treatment, which can reduce quality of life and cause a serious social burden [9]. 

Wheat allergens are divided into two groups: gluten proteins and non-gluten proteins. Non-gluten proteins are further divided into albumins (water-soluble) and globulins (salt-soluble), which have metabolic and structural functionalities [10]. Gluten proteins are seed storage proteins that are constituted by gliadins and glutenins. Gliadins are prolamin proteins that are ethanol-soluble, and glutenins are glutelin proteins that are soluble in weak acid (acetic acid) solution [11]. Both non-gluten and gluten proteins are linked to wheat hypersensitivity (or allergy) in humans [12,13].

The development of wheat allergy comprises two consecutive stages: (1) the production of IgE antibodies against specific wheat allergens upon initial encounters with the allergen causing sensitization; (2) Triggering of allergic response upon subsequent exposure to wheat allergens eliciting potentially life-threatening anaphylaxis. It is generally accepted that oral consumption of dietary gluten may cause sensitization [11]. There is also suggestion that skin, airways, and eyes exposure may be involved in sensitization [8,14,15,16]. Currently, it is unknown whether wheat glutenin (WG) has intrinsic sensitization capacity, or whether skin exposure to WG can cause clinical sensitization for life-threatening systemic anaphylaxis.

Several animal models (dog, rat, mice) have been used to study wheat allergenicity using gluten and non-gluten proteins [17,18,19,20,21,22,23]. Wheat gliadin has been used in most gluten allergy mouse model studies [20,22,24,25]. There are two mouse models reported for wheat glutenin hypersensitivity [19,26]. While these models are very useful with their own strengths and novelty, there are few major challenges that limit their applications, such as: (i) use of adjuvants to elicit sensitization to glutenin; (ii) exposure to glutenin via injections to elicit sensitization; and (iii) lack of robust quantitative readouts of systemic anaphylaxis (e.g., hypothermia shock response, and mediators). In this study, we sought to address these limitations, thereby further refining and improving the animal model of glutenin hypersensitivity.

We used a transdermal sensitization method to develop this mouse model of wheat glutenin allergy because: (i) there is evidence that humans can be sensitized to food allergens including glutenin allergens via skin exposure [27,28,29]; for example there are reports of developing wheat glutenin sensitization after using facial soaps in Japan [30,31,32]; and (ii) in mouse models, oral exposure to dietary proteins results in immune tolerance unless adjuvants such as cholera toxin, etc., are co-administered. Our intent was to develop an adjuvant-free mouse model of systemic anaphylaxis, therefore, to bypass oral immune tolerance, we used a transdermal route of exposure without an adjuvant to induce sensitization.

We used glutenin to develop this mouse model because: (i) glutenin proteins are associated with sensitization as well as life-threatening anaphylaxis in humans, and therefore, a mouse model that could be used to study wheat glutenin-induced sensitization and anaphylaxis would help clarify mechanisms as well as develop novel preventative and therapeutics; (ii) facial soaps containing glutenin have been reported to induce sensitization for systemic anaphylaxis in humans [33,34]; (iii) the gluten family of proteins are classified into two distinct groups: gliadin (30–40% of total wheat protein) and glutenin (45–50% total wheat proteins) [11,35]; although there are several mouse models of anaphylaxis reported for gliadins [21,22,24], there are only two mouse models (adjuvant and injection-based) reported for glutenin sensitization [19,26]; however, an adjuvant-free mouse model simulating skin exposure to glutenin leading to sensitization and anaphylaxis is unavailable at present, and such a model would be very useful in basic and applied research on wheat glutenin allergy. 

In this study, we tested the hypothesis that repeated skin exposures to WG extract without adjuvant will sensitize mice with the resultant anaphylactic reaction upon intraperitoneal WG challenge. There were six objectives for this research: (1) establish a colony of plant protein-free Balb/c mice colony; (2) Assess WG’s inherent sensitization potential (i.e., IgE response) via repeated skin application; (3) investigate anaphylactic clinical symptoms upon systemic WG challenge; (4) Quantify the anaphylaxis using hypothermic shock responses (HSR); (5) Measure mucosal mast cell degranulation responses (MMCR) by quantifying blood levels of MMCP-1; and (6) Identify spleen biomarkers associated with life-threatening systemic anaphylaxis in this model. Overall, these data reveal for the first time that WG is intrinsically allergenic, and that chronic skin exposure to WG can prime the mice for potentially fatal anaphylaxis. Life-threatening anaphylaxis is associated with differential expression of immune biomarkers involved in vascular permeability, and allergic immune regulation.

## 2. Results

### 2.1. Chronic Application of Wheat Glutenin (WG) onto Undamaged Skin Elicits Robust Specific IgE, and IgG1 Antibody Response in Balb/c Mice

**Figure 1:**
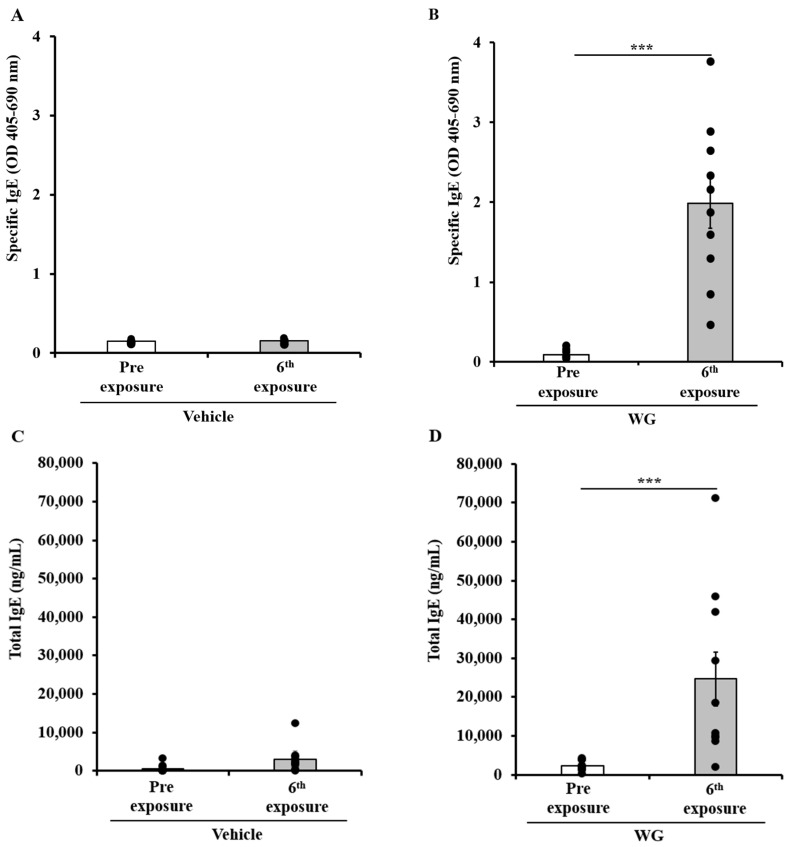
Chronic skin exposure to wheat glutenin (WG) extract elicited specific IgE antibody responses and elevation of total IgE in Balb/c mice. Mice were exposed to WG or to vehicle, as described in the Materials and Methods section. Blood was collected before first exposure (Pre) and after sixth exposure. Plasma was used in measurement of WG-specific IgE levels (OD 405–690 nm) using an ELISA method described previously. Each dot represents one mouse data. (**A**) WG-specific IgE antibody levels in control mice. (**B**) WG-specific IgE antibody levels in sensitized mice. (**C**) Total IgE levels in vehicle sensitized control mice. (**D**) Total IgE levels in WG-sensitized mice. Student’s two-tailed *t*-test: *** *p* < 0.001.

The potential of WG to induce sensitization when applied repeatedly to the skin was conducted as follows: Female adult Balb/c mice were divided into groups (*n* = 10/group) and subjected to topical application of either WG or control vehicle once per week for nine weeks, as described in the methods section. Blood samples were collected before the initial exposure (pre) and after six skin exposures using a pre-optimized ELISA method to quantify specific (s) IgE levels. As depicted in Figure 1A,B, skin exposure to WG resulted in a significant sIgE response, whereas the vehicle displayed no such elevation. The WG elicited IgE antibodies did not find to other irrelevant allergens (Supplemental Figure S1). In addition to IgE, skin exposure to WG also elicited IgG1 antibody response (Supplemental Figure S2). 

### 2.2. Chronic Application of Wheat Glutenin (WG) onto Undamaged Skin also Elevates Total IgE Levels, Which Correlate with sIgE Levels

**Figure 2:**
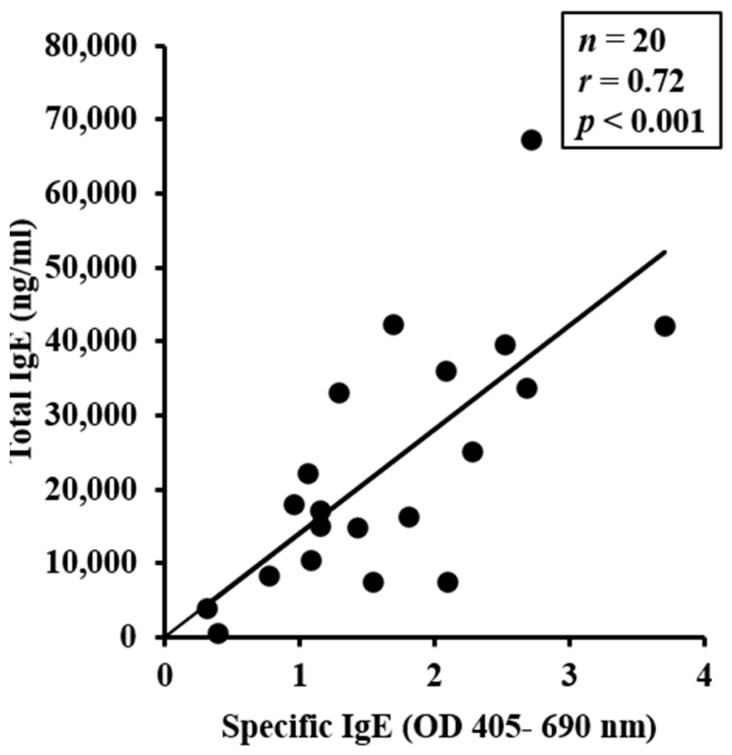
Pearson correlation analysis between wheat glutenin (WG)-specific IgE antibody levels and total IgE levels. Mice were treated as described in the Materials and Methods section. Pearson correlation analysis was used to test the relationship between WG-specific IgE antibody and total IgE levels in the plasma after 6th transdermal exposure to WG. Each dot represents one mouse data.

Total (t)IgE levels in the blood were assessed using an ELISA method before (pre) and after six exposures to either WG or to vehicle. As evident, prolonged exposure to WG significantly elevated tIgE levels, which was not observed in mice exposed to the vehicle (Figure 1C,D). To examine the relationship between individual mouse data for sIgE and tIgE, Pearson correlation coefficient analysis was performed. A significant positive correlation between the two measurements was observed (Figure 2). 

### 2.3. WG-Sensitized, but Not Vehicle-Sensitized Mice, Exhibit Life-Threatening Symptoms of Anaphylaxis upon Systemic Challenge with WG

**Figure 3:**
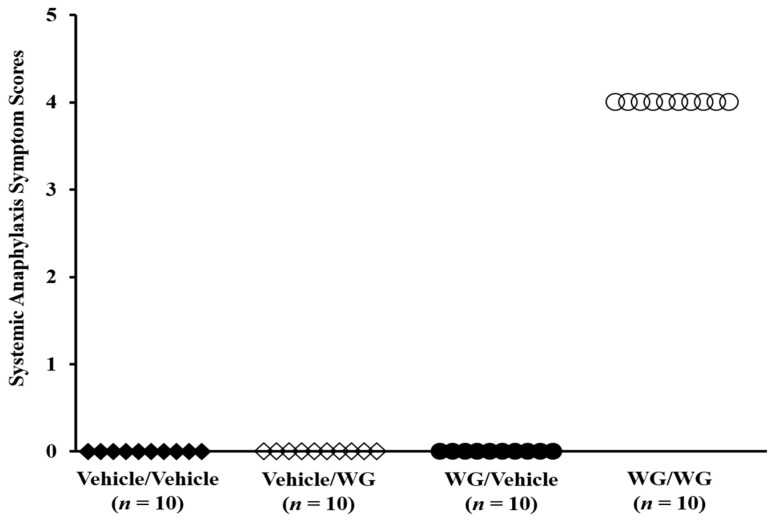
Chronic skin exposure to wheat glutenin (WG) is sufficient to clinically sensitize Balb/c mice for systemic anaphylaxis. Groups of Balb/c mice were sensitized with either vehicle or WG as described in the Methods section. After nine transdermal exposures mice were challenged with either vehicle or WG (0.5 mg/mouse) by intraperitoneal injection. Each symbol represents an individual mouse.

Two separate sets of mice, one sensitized with WG and the other sensitized with vehicle, were challenged with intraperitoneal WG to assess the presence of systemic anaphylaxis. Clinical symptoms were assessed using established methods [36]. Notably, life-threatening clinical symptoms were observed exclusively in the WG-sensitized mice, but not in the vehicle-exposed groups (Figure 3). The most prevalent symptoms included altered respiration, scratching, rubbing of the nose, face, and/or head, and lack of activity upon prodding. 

### 2.4. Mice Experiencing Systemic Anaphylaxis Symptoms Following a Systemic Challenge with WG Displayed Pronounced Hypothermic Shock Responses (HSR)

**Figure 4:**
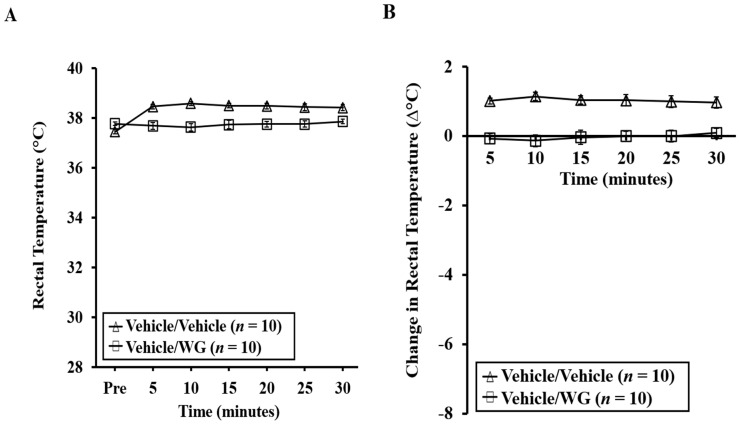

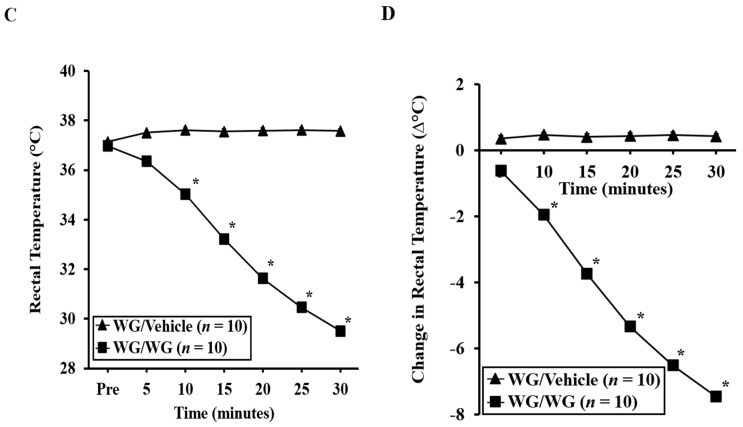
Induction of hypothermic shock responses upon systemic challenge with wheat glutenin (WG). Mice exposed to WG or to vehicle were systemically challenged by intraperitoneal injection as described in the Materials and Methods section. (**A**) Rectal temperatures (°C) at indicated time points in vehicle-sensitized mice challenged with WG or vehicle. (**B**) Change in rectal temperature (∆°C) at indicated time points in vehicle-sensitized mice challenged with WG or vehicle. (**C**) Rectal temperatures (°C) at indicated time points in WG-sensitized mice challenged with WG or vehicle. (**D**) Change in rectal temperature (∆°C) at indicated time points in WG-sensitized mice challenged with WG or vehicle. * *p* < 0.05.

Anaphylactic reactions were further assessed using rectal thermometry to examine hypothermic shock responses (HSR). It is evident that systemic challenge with WG led to life-threatening HSR in WG-sensitized mice, while no such response was observed in the vehicle control mice (Figure 4A–D). Actual temperature changes are depicted in Figure 4A,C. Absolute temperature changes are shown in Figure 4B,D. Notably, no HSR was observed in mice challenged with vehicle, or in mice sensitized with vehicle and subsequently challenged with WG. 

### 2.5. Systemic Anaphylaxis Is also Linked to Substantial Mucosal Mast Cell Degranulation in This Model

**Figure 5:**
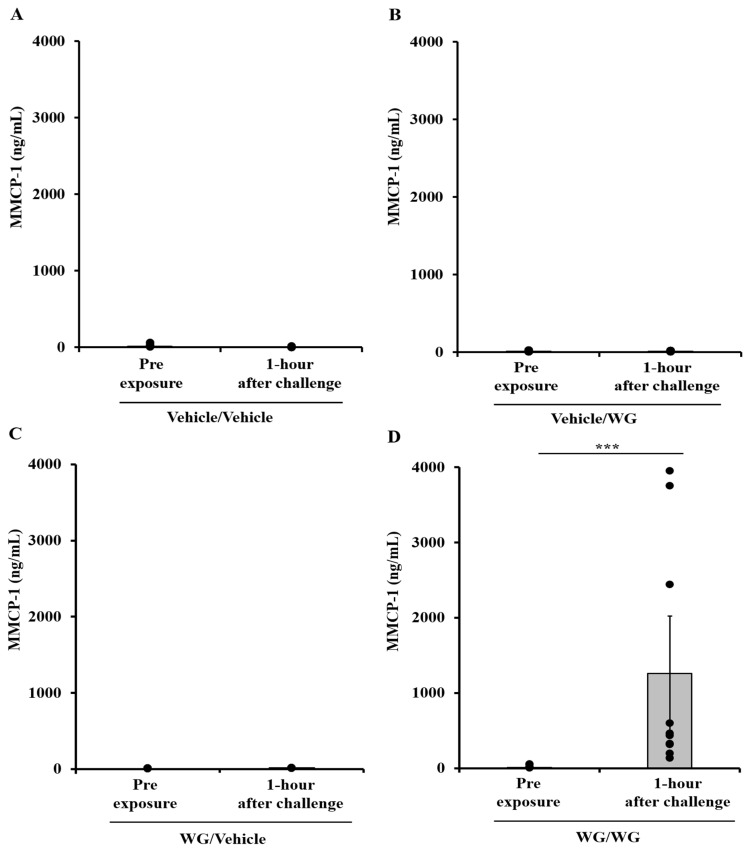
Systemic anaphylaxis induced by wheat glutenin (WG) is associated with degranulation of mucosal mast cells in this model. Mice were treated as described in the Materials and Methods section. Their plasma mucosal mast cell protease-1 (MMCP) levels (ng/mL) were measured using an ELISA-based method described in the texts. (**A**) MMCP-1 levels in control mice challenged with vehicle. (**B**) MMCP-1 levels in vehicle-sensitized control mice challenged with WG. (**C**) MMCP-1 levels in WG-sensitized mice challenged with vehicle. (**D**) MMCP-1 levels in WG-sensitized mice challenged with WG. Each dot represents one mouse data. Student’s two-tailed *t*-test: *** *p* < 0.001.

Blood samples were obtained one hour after intraperitoneal challenge and utilized to assess the mucosal mast cell degranulation response (MMCR) in mice. The increase in mucosal mast cell protease-1 (MMCP-1) serves as evidence of a genuine IgE antibody-mediated type-1 hypersensitivity reaction to food proteins in mouse models, as described previously [37]. Figure 5A–D clearly demonstrates a significant MMCR in mice undergoing anaphylaxis, while no such MMCR was observed in the control mice. 

### 2.6. Proteomic Analysis and Identification of Differentially Expressed Immune Biomarkers in the Spleen of Mice Undergoing Systemic Anaphylaxis 

**Figure 6:**
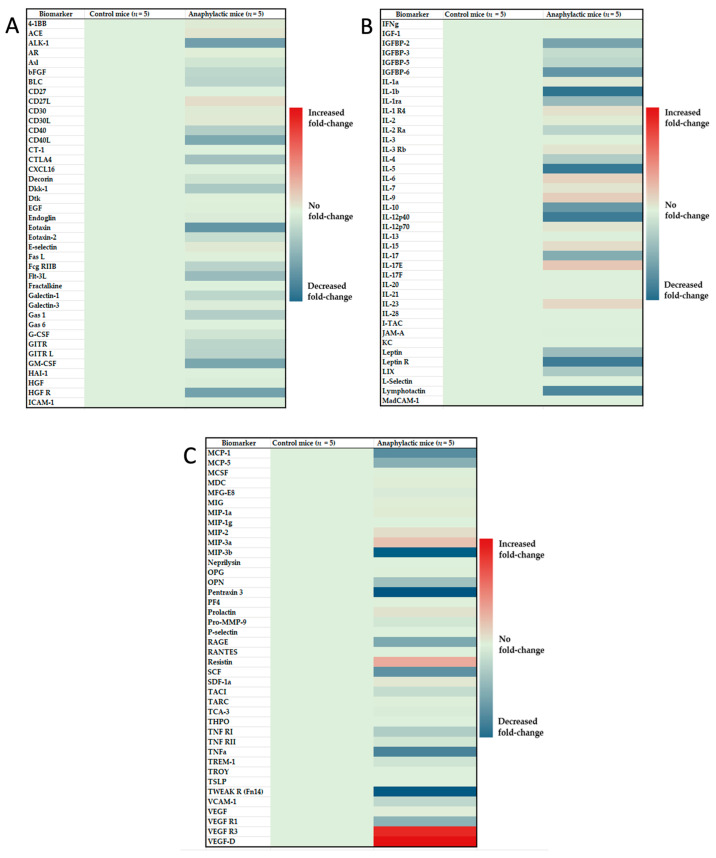
Heat map analysis of 120 spleen immune biomarkers in glutenin-induced systemic anaphylaxis. Using spleen extracts from control mice and anaphylactic mice, a proteomic microarray analysis was conducted using RayBiotech system cytokine panels, (**A**) CYT-4, (**B**) CYT-5 and (**C**) CYT-6 as described in the methods. Background levels of immune biomarkers are shown in green. Upregulated biomarkers are shown in red, and down-regulated biomarkers are shown in blue.

We conducted a heat map analysis of the expression of a panel of 120 proteomic immune biomarkers in mice undergoing anaphylaxis versus the control mice, as described in methods (Figure 6A–C). Among the differentially expressed immune biomarkers, 27 markers were significantly elevated and 37 were significantly reduced (Student’s *t*-test, two-tailed, *p* < 0.05) in anaphylactic mice (Table 1 and Table 2). 

**Figure 7:**
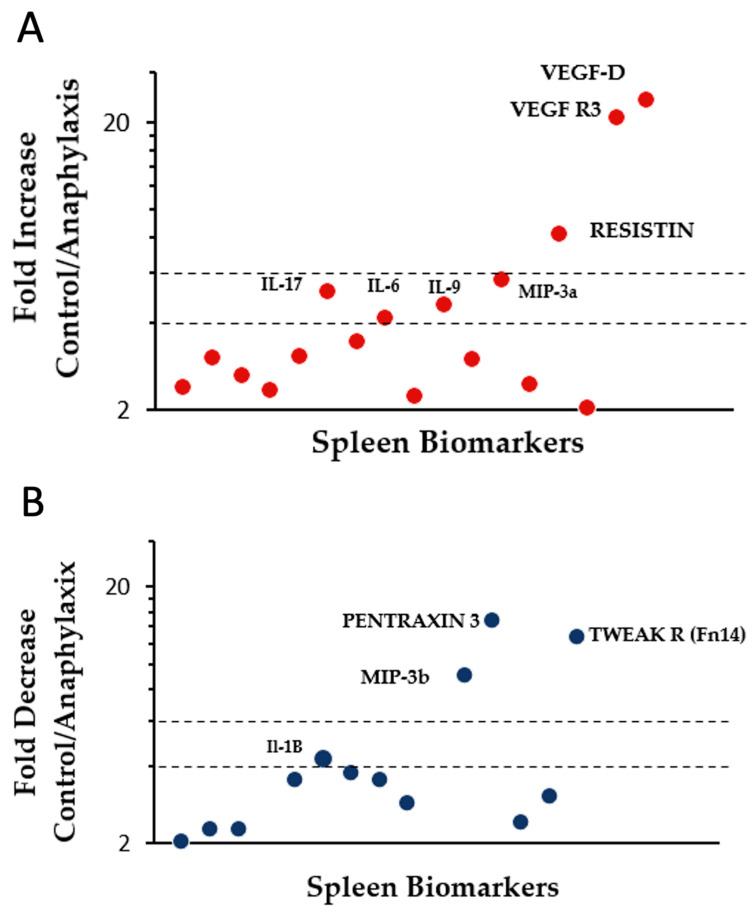
Identification of differentially expressed immune biomarkers in the spleen of control vs. anaphylactic mice. (**A**) Immune biomarkers that are significantly elevated (2-fold or higher, *p* < 0.05) in anaphylactic mice are shown. (**B**) Immune biomarkers that are significantly decreased (2-fold or lower, *p* < 0.05) in anaphylactic mice are shown. The dotted lines indicate a 4-fold and 6-fold changes in protein expression. Immune biomarkers that show 4-fold or higher changes are identified with names.

Next, we classified these makers into four categories based on fold-change in protein expression as follows: low importance (up to 1.9-fold change), medium importance (2–3.9-fold change), high importance (4–5.9-fold change), and critical importance (6 and above-fold change). The following seven immune biomarkers were substantially elevated in anaphylaxis: IL-6, IL-9, IL-17E, and MIP-3a (high importance), Resistin, VEGF-D, and VEGF-R3 (critical importance) (Figure 7A). The following four immune biomarkers were markedly reduced in anaphylaxis: IL-1b (high importance), MIP-3b, Pentraxin 3, and TWEAK R (critical importance) (Figure 7B).

## 3. Discussion

The primary objective of this study was to determine whether wheat glutenin is intrinsically allergenic in mice. It is currently unknown whether wheat glutenin by itself, in the absence of adjuvants such as alum or complete Freund’s adjuvant, etc., is capable of sensitizing animals for clinical elicitation of systemic anaphylaxis. Therefore, in this study, we tested the hypothesis that wheat glutenin will clinically sensitize mice upon transdermal application to glutenin without any external adjuvants. Our data collectively support this hypothesis. 

There are eight novel findings: (i) Chronic application of glutenin onto undamaged skin elicits robust specific IgE antibody response in Balb/c mice in an exposure dependent fashion; (ii) chronic application of glutenin onto undamaged skin also elevates total IgE levels, which correlate with sIgE levels; (iii) mice that were glutenin-sensitized, but not vehicle-sensitized exhibit life-threatening symptoms of anaphylaxis upon systemic challenge with glutenin, but not with vehicle; (iv) mice with systemic anaphylaxis symptoms upon intraperitoneal challenge with glutenin exhibit dramatic and life-threatening hypothermic shock responses (HSR); (v) HSR was associated with significantly elevated mucosal mast cell response as quantified by MMCP-1 levels in the plasma; (vi) identification of differentially expressed immune biomarkers by heat map analysis in the spleen of anaphylactic vs. control mice; (vii) identification of biomarkers positively and negatively associated with anaphylaxis compared to healthy mice based on significant fold-change in protein expression; and (viii) identification of biomarkers of high and critical importance that are substantially altered in mice during life-threatening anaphylactic reaction compared to healthy control mice in this model. 

In this study we chose to establish a mouse model of glutenin for the following reasons: (i) it is unknown at present whether wheat glutenin has the intrinsic capacity to elicit IgE antibody responses and whether it can cause systemic anaphylaxis in the absence of exercise as a cofactor; (ii) it is unknown at present whether wheat glutenin skin exposure can clinically sensitize mice for systemic anaphylaxis; (iii) most of the previous mouse models of wheat hypersensitivity have used wheat gliadins for developing the models; (iv) there are only two previous mouse model studies, both of which used adjuvants to elicit IgE responses to glutenin [19,26]. 

Kozai et al., 2006, reported the first mouse model to elicit IgE antibody responses to wheat glutenin [19]. Strengths of their model include: (i) this was the first model of glutenin-dependent exercise-induced exhaustion reported in the literature; (ii) these authors elegantly demonstrated that sensitization to glutenin by IP injections with alum followed by oral glutenin challenge (20 mg/mouse) results in significantly reduced time to exhaustion upon being subjected to exercise using a treadmill. The limitations of the study include: (i) indicators of anaphylaxis, such as clinical symptom scores, hypothermic shock responses, histamine responses, or mucosal mast cell mediator proteins were not reported; therefore, whether IgE antibody response resulted in clinical sensitization for systemic anaphylaxis was not studied; (ii) this was a complete Freund’s adjuvant-based model to elicit sensitization; therefore, intrinsic sensitization capacity of wheat glutenin was not studied. 

Wang et al., 2020, reported a mouse model of glutenin sensitization using alum adjuvant-based method [26]. Strengths of their model include: (i) demonstration of IgE antibody responses to glutenin injection with alum; (ii) demonstration of clinical symptoms of anaphylaxis by one hour after intragastric challenge with glutenin (20 mg/mouse). Limitations of this study include: (i) use of alum adjuvant to elicit sensitization to wheat glutenin; therefore, intrinsic sensitization capacity of glutenin was not studied; (ii) hypothermic shock responses, which are widely used as a quantitative indicator of systemic anaphylaxis, were not studied; (iii) immediate hypersensitivity mediators were not studied; responses were studied at 24 h post intragastric challenge, which does not reflect the immediate hypersensitivity response that is well established to occur within one hour post challenge [37].

Other animal models have been reported for gluten allergy, such as Buchanon et al., 1997, which utilized a dog model of food allergy to multiple foods, including wheat [18]. In this model, genetically selected dogs were used for sensitization to wheat flour along with alum adjuvant. In addition, distemper and hepatitis vaccination was also administered. Dogs developed immediate hypersensitivity reactions to both gluten allergens (gliadin and glutenin) as well as non-gluten allergens (albumin and globulin), as evidenced by positive skin prick test reactions. Strengths of this model include: (i) this was the first animal model demonstrating sensitization to wheat glutenin when injected with alum adjuvant; (ii) oral challenge with wheat flour gruel elicited diarrhea, indicating oral food allergic reaction. Limitations of this study include: (i) use of alum adjuvant does not allow for the investigation of the intrinsic allergenicity of the wheat glutenins; (ii) it is a model of oral wheat-induced diarrhea, and the dogs did not develop life-threatening anaphylactic reactions; (iii) they did not report characterization of IgE antibody responses to glutenin. 

We conducted a proteomic analysis of spleen immune biomarkers and identified differentially expressed immune biomarkers in mice undergoing near-fatal systemic anaphylaxis versus healthy control mice using a large panel (120) of biomarkers implicated in immune and inflammatory responses. Among them, 27 biomarkers were significantly upregulated, and 37 biomarkers were significantly downregulated during anaphylaxis. These markers have been linked to inflammation, immune regulation, and airways allergic responses in mouse models and in humans [38,39,40,41,42,43,44,45]. However, we demonstrate the differential expression of immune biomarkers associated positively and negatively with glutenin-induced life-threatening anaphylaxis compared to healthy control mice for the first time. 

Based on fold-change analyses, we identified four immune markers (IL-6, IL-9, IL-17E, MIP-3a) that were of high importance (4–5.9-fold or higher) during anaphylaxis. Consistent with our findings here, both IL-17E and MIP-3a have been implicated in the pathogenesis of allergic immune responses previously, although linkage to glutenin-induced anaphylaxis is a novel finding [45,46]. Previously, IL-9 has been found to be associated with anaphylaxis induction [47]. Three biomarkers were determined to be of critical importance (6-fold or higher) during anaphylactic reactions (resistin, VEGF R3, and VEGF-D). Previous studies have reported a conflicting role of serum resistin levels in mouse-models of airways allergies [48]. In this study, we demonstrate a potential role for resistin in glutenin-induced systemic anaphylaxis for the first time. Two cytokines important in vascular permeability (VEGF R3, VEGF-D) were also dramatically elevated, which is consistent with the concept of life-threatening uncontrolled vascular leakage of fluid during anaphylactic shock [49,50]. Therefore, these cytokines may represent potential targets for modulating vascular permeability during anaphylaxis. 

Interestingly, there were four biomarkers whose expression was markedly reduced during anaphylaxis. Of these, we identified one biomarker that is of high importance (IL-1B), and three others (MIP-3b, TWEAK R, and Pentraxin 3) that were of critical importance during anaphylaxis. There are previous studies that propose a protective role for Pentraxin 3 and MIP-3b in airways allergies [51,52]. Our findings regarding their negative association with anaphylaxis expands their potential protective roles beyond airways allergic reactions to glutenin-induced life-threatening systemic anaphylaxis. Notably, the TWEAK R (Fn14) axis has been positively linked to anaphylaxis in previous mouse models of passive and active sensitization with adjuvant [53,54]. In this study, we demonstrate for the first time that TWEAK R is negatively linked to glutenin-induced active anaphylaxis in an adjuvant-free mouse model of food allergy. This discrepancy is discussed below. 

In our adjuvant-free mouse model of glutenin-induced systemic anaphylaxis, we found that TWEAK R protein levels in the spleen tissue was critically reduced in anaphylactic mice. This finding contrasts with a previous report that TWEAK R expression was significantly elevated in the lung tissue in an adjuvant-based mouse model of anaphylaxis and in an anti-hapten IgE antibody sensitized passive systemic anaphylaxis model [55]. This discrepancy may be due to differences between our model and their models as follows: (i) they used C57/BL 6 mice sensitized passively with anti-hapten (DNP) IgE antibodies, followed by intravenous injection with hapten-carrier complex to elicit passive systemic anaphylaxis. They also used C57/BL 6 mice sensitized with BSA (bovine serum albumin) plus pertussis toxin adjuvant via intraperitoneal injection, followed by active systemic anaphylaxis induced by intravenous injection with BSA; in contrast, we used Balb/c mice sensitized with WG without adjuvant via skin application, followed by intraperitoneal injection with WG to elicit active systemic anaphylaxis; (ii) they studied TWEAK R expression and reported differences in staining intensity of lung tissue section by immunohistochemistry; in contrast, we quantified TWEAK R protein absolute concentration in the spleen tissue extract using a quantitative method in our model. These discrepancies suggest that molecular characteristics of anaphylaxis may be different in different models and tissues. 

In this report, spleen immune biomarkers were studied in healthy control mice and in anaphylactic mice. Therefore, at least three follow-up studies are needed to establish the anaphylaxis specificity of the differentially expressed biomarkers reported in this study: (i) to determine the contribution of sensitization (in the absence of anaphylaxis) to observed changes in expression of biomarkers in the spleen; (ii) to determine whether WG injection to unsensitized healthy mice impact the expression of immune biomarkers in the spleen; and (iii) to determine tissue-specific changes in the expression of these immune biomarkers during anaphylaxis in this mouse model. 

In this study, we have significantly advanced the animal model development for glutenin hypersensitivity compared to previously existing models, as discussed above. In particular, several major limitations of previous models were addressed in our model, as follows: (i) we report a novel adjuvant-free animal model of glutenin hypersensitivity that can be used to assess intrinsic allergenicity potential of wheat glutenin; (ii) we demonstrate that chronic skin exposures to glutenin without causing deliberate damage (for example, tape-stripping of stratum corneum, an approach commonly used to develop skin sensitization rodent models); (iii) we not only characterized clinical symptom scores of systemic anaphylaxis, but advanced them further by developing a robust quantifiable method, such as hypothermic shock responses; (iv) we report a robust immediate (at one-hour) mucosal mast cell degranulation response upon systemic challenge with glutenin; (v) using single mouse data analysis, we demonstrate a significant correlations between two common clinical indicators of sensitization (specific IgE, total IgE); and (vi) we also identified several systemic immune markers associated with life-threatening anaphylaxis compared to healthy mice. 

Systemic anaphylaxis upon allergen injection (intraperitoneal, intravenous) in mouse models can be mediated by allergen specific IgG1 antibodies [55,56]. Therefore, we measured WG-specific IgG1 antibody responses. Results show that WG elicits a robust IgG1 anti-body response in this model. Thus, WG-specific IgG1 antibodies may also contribute to systemic anaphylaxis upon intraperitoneal injection in this model, and this may represent a limitation of this study. 

This study was conducted using glutenin obtained from hexaploidy wheat (*Triticum aestivum*, ambassador variety, genome AABBDD). Therefore, this model can be used to compare intrinsic allergenicity of glutenin from other genetically distinct wheats available on AABBDD genome as well as other wheats with different ploidy, such as AA (*Triticum monococcum*), AABB (*Triticum durum*), and DD (*Aegilops tauschii*). Any novel wheat developed with these genomes as their background can also be preemptively tested for their intrinsic allergenicity of glutenins. 

Currently, genetically engineered (GM) wheat is not commercially available. However, there are efforts to develop them. For example, the US FDA approved a GM wheat developed by Argentina [57]. There were previous incidents of GM wheat contamination of US and Canadian farms that were investigated [58]. It would be critical to address potential allergenicity concerns about GM wheat. International scientific organizations (WHO/FAO) have provided a decision tree approach to evaluate the allergenicity hazard of novel GM wheats [59]. They have suggested using validated animal models for testing. The model that we have described here would be very useful for preclinical evaluation of intrinsic allergenicity of glutenins obtained from such GM wheats. 

This model can be used to develop novel immunotherapies to wheat glutenin allergy. For example, wheat glutenin-sensitized mice can be used to test a novel protocol, such as repeated low-dose oral administration of native or modified wheat glutenin to desensitize mice from wheat glutenin allergy. In the same way, novel drugs can be developed for glutenin-induced life-threatening systemic anaphylaxis using this model for pre-clinical testing. 

Food processing methods have been shown to influence (increase/decrease/eliminate) wheat allergenicity in in vitro methods [60]. This model provides an opportunity to determine the effects of various physical, chemical, microbiological processing methods on glutenin allergenicity and aid in development of potentially hypo/non-allergenic glutenin proteins. Similarly, inadvertent creation of hyper-allergenic and dangerous glutenins can be prevented by preemptive testing using this model. 

In summary, this study collectively reveals wheat glutenin’s intrinsic allergenic nature for the first time in an animal model. This model also provides robust quantifiable readouts of life-threatening systemic anaphylaxis. Therefore, this improved model of glutenin allergenicity can be utilized to develop novel methods to prevent and treat life-threatening anaphylactic reactions to glutenin in humans. 

## 4. Materials and Methods

### 4.1. Chemicals and Reagents

Biotin-conjugated rat anti-mouse IgE-paired antibodies were procured from BD BioSciences (San Jose, CA, USA). The p-nitro-phenyl phosphate compound was sourced from Sigma (St. Louis, MO, USA). Streptavidin alkaline phosphatase was acquired from Jackson ImmunoResearch (West Grove, PA, USA). Folin reagent was obtained from BioRad (Hercules, CA, USA). The following reagents were secured as specified: IgE Mouse Uncoated ELISA Kit with Plates, Streptavidin-HRP, TMB substrate, MCPT-1 (mMCP-1) Mouse Uncoated ELISA Kit with Plates, Avidin-HRP, TMB substrate—all of which were procured from Invitrogen (Waltham, MA, USA). The Tissue Protein Extraction Reagent (T-PERTM), a proprietary detergent with a composition of 25 mM bicine and 150 mM sodium chloride at pH 7.6, was obtained from ThermoFisher Scientific (Waltham, MA, USA). For protease inhibition, a cocktail of serine, cysteine, and acid proteases, along with aminopeptidases, was acquired from Sigma-Aldrich (St. Louis, MO, USA).

### 4.2. Mice Breeding and Establishment of a Plant-Protein-Free Mouse Colony

Adult Balb/c breeder pairs were obtained from The Jackson Laboratory (Bar Harbor, ME, USA). Upon arrival, the mice were introduced to a rigorous plant-protein-free diet (AIN-93G, Envigo, Madison, MI, USA). Following a one-week acclimation period, breeding was initiated using conventional methods. For this study, adult female mice aged 6–8 weeks from the litter were selected. Throughout the entire duration of the study, all mice were consistently maintained on the strict plant-protein-free diet (AIN-93G). All animal procedures adhered to the guidelines outlined by Michigan State University (AMEND202200325 PROTO202100331).

### 4.3. Preparation of Acid-Soluble Protein Extract from Wheat Flour

Hexaploid wheat flour was used for protein extraction purposes. The acid-soluble wheat glutenin was obtained through an Osborne sequential extraction method [61]. In brief, a mixture of flour and filter-sterilized 0.5 M NaCl at a ratio of 1:10 (*m*/*v*) was continuously agitated for 2 h and then subjected to centrifugation at 20,000× *g* for 30 min. The resultant pellets were preserved and utilized for alcohol extraction. The salt-insoluble pellets were subsequently mixed in a 1:10 ratio with 70% ethanol for 2 h and then centrifuged at 20,000× *g* for 15 min. The resultant pellets (alcohol-insoluble) were preserved to be used in acid extraction. The alcohol-insoluble pellets were combined in a 1:4 ratio with 0.05 M acetic acid for two hours, then centrifuged at 20,000× *g* for 15 min. The resulting supernatant was frozen at −70 °C overnight and then subjected to freeze-drying the following day. The lyophilized acid-soluble wheat glutenin (WG) was reconstituted using 0.05 M acetic acid to achieve a concentration of 1 mg protein per 100 µL, intended for topical application. For challenges involving intraperitoneal (IP) injections, the WG was reconstituted with phosphate buffered saline (PBS) to attain concentrations of 0.5 mg/mouse. The protein content was quantified using the LECO total combustion method from LECO (St. Joseph, MI, USA). To assess protein quality, SDS-PAGE testing was performed.

### 4.4. Skin Sensitization, Bleeding, and Plasma Sample Preparation

Female adult Balb/c mice were employed for experimental purposes. To facilitate the procedures, the hair on the mice’s rumps was removed bilaterally using a Philips hair clipper (Amsterdam, The Netherlands). The acid-soluble wheat glutenin (WG) was administered onto the rump at a dosage of 1 mg per mouse in 100 µL, or alternatively, using a vehicle solution of 0.05 M acetic acid. Following application, the treated area was covered with a non-latex bandage sourced from Johnson & Johnson (New Brunswick, NJ, USA), which was left in place for one day. This process was reiterated on a weekly basis, occurring nine times over a span of nine weeks. Blood samples were collected from the saphenous vein prior to the initial exposure and after the sixth exposure. The blood was drawn into tubes coated with the anticoagulant lithium heparin (Sarstedt Inc., MicrovetteCB 300 LH, Numbrecht, Germany). The collected blood was subsequently subjected to centrifugation to isolate plasma, which was then stored individually at −70 °C until required for subsequent testing of (s)IgE and (t)IgE. 

### 4.5. Elicitation of Systemic Anaphylaxis and Clinical Symptom Scoring

Two weeks after the final cutaneous exposure to acid-soluble wheat glutenin (WG) or the vehicle, the mice were subjected to an intraperitoneal (IP) injection. This injection consisted of either 0.5 mg of WG or the vehicle (phosphate-buffered saline, PBS). Following the injection, the mice were closely monitored for signs of systemic anaphylaxis over a 30 min period, in accordance with previously outlined protocols [22,37]. Assessment scores were assigned based on the ensuing criteria: 0 indicated an absence of symptoms; 1 denoted behaviors like nose and head scratching, along with rubbing; a score of 2 encompassed observations such as swelling around the eyes and mouth, diarrhea, erection of hair (pilar erecti), reduced activity, and/or lowered activity coupled with an elevated respiratory rate; 3 was attributed to manifestations like wheezing, labored breathing, and bluish discoloration near the tail and mouth; 4 marked a lack of activity even after stimulation, accompanied by tremors and convulsions; and, ultimately, 5 indicated mortality.

### 4.6. Determination of Hypothermic Shock Responses

Rectal temperature (°C) measurements were taken both prior to the challenge and at 5-min intervals following the challenge, up to a 30-min duration. These measurements were conducted using a rectal thermometer (DIGI-SENSE, Vernon Hills, IL, USA). The recorded values included the specific temperatures and the corresponding differences (∆°C) in comparison to the pre-challenge temperatures for each individual mouse. These recorded data points were then employed for subsequent analyses.

### 4.7. Measurement of Specific IgE Antibody Levels

WG-specific (s) IgE antibody levels were quantified using a highly sensitive ELISA method, as previously detailed with certain modifications [14,22,62,63]. Initially, 96-well Corning 3369 plates were coated with WG and, subsequently, blocked using a 5% gelatin solution. After a thorough washing step, plasma samples were introduced onto the plate. Further washing ensued, followed by the addition of a biotin-conjugated anti-mouse IgE antibody. Subsequent washes were performed before introducing streptavidin alkaline phosphatase, and eventually, p-nitro-phenyl phosphate were added to enable quantification, mirroring established methodologies [14,22,63]. 

### 4.8. Measurement of Total Plasma IgE Concentration

Total IgE concentrations were determined utilizing an Invitrogen ELISA kit (Waltham, MA, USA). Briefly, 96-well Corning Costar 9018 plates were coated with anti-mouse IgE capture antibodies with the subsequent addition of standards and plasma samples (recombinant mouse IgE). Anti-mouse IgE was utilized as a secondary antibody followed by a detection system of streptavidin-HRP and TMB substrate as described by previous studies [14,63]. The assay limit of detection is 4 ng/mL. The standard range for the analysis was 4–250 ng/mL. 

### 4.9. Quantification of Mucosal Mast Cell Protease-1 (MMCP-1) Level

Blood samples were collected one hour after the challenge and utilized for quantifying mucosal mast cell protease-1 (MMCP-1) levels in the plasma. This measurement was conducted using an ELISA-based approach developed by Invitrogen, consistent with previously outlined procedures [14,63]. To elaborate, 96-well Corning Costar 9018 plates were initially coated with a capture antibody (anti-mouse MMCP-1). Subsequently, samples and standards (recombinant mouse MMCP-1) were introduced onto the plate. Biotin-conjugated anti-mouse MMCP-1 antibody was then added as the secondary antibody. Detection was accomplished through the utilization of an avidin-HRP/TMB substrate system. Notably, the assay possesses a limit of detection set at 120 pg/mL, and the range of standards spanned from 120 to 15,000 pg/mL. Each individual mouse’s plasma was subjected to testing in quadruplicate.

### 4.10. Spleen Extract Preparation and Proteomic Analysis of Immune Biomarkers

One hour after the challenge, mice were humanely euthanized, and their spleens were procured. The harvested spleens were promptly frozen in liquid nitrogen and preserved at −70 °C. The tissue extraction process followed previously established procedures [22,62]. To elaborate, the spleen tissue was immersed in a tissue protein extraction reagent (T-PER) buffer that contained protease inhibitor. For every 100 mg of tissue, a proportion of 10 µL of protease inhibitor per 1 mL of T-PER buffer was utilized. Homogenization of the spleen tissue was achieved through ultrasonication, performed over two 30-s cycles with an intervening rest period of 5 min. After the second homogenization step, the samples were allowed to rest for 15 min and then subjected to centrifugation at 13,500× *g* at 4 °C for 10 min. The resulting supernatant was meticulously collected and divided into aliquots for storage at −70 °C. For quantification of immune markers, Quantibody microarray (CYT-4, 5, and 6, 120 marker panel involved in inflammation, immune regulation, and hypersensitivity) was employed (RayBiotech, Atlanta, GA, USA). This array allowed for the assessment of immune markers. The analysis was conducted in quadruplicate for each sample.

### 4.11. IgG1 

WG-specific IgG1 antibody levels were measured using a modified ultrasensitive ELISA method as previously reported [22,64]. Next, 96-well Corning 3369 plates were coated with WG and blocked with bovine serum albumin. The plates were then washed, and plasma samples were added. After a subsequent washing step, biotin-conjugated anti-mouse IgG antibodies were added, followed by additional washes. Streptavidin alkaline phosphatase was then added, and p-nitro-phenyl phosphate was used for quantification, as described previously [22,64].

### 4.12. IgE Cross-Reactivity

Allergen-specific (s) IgE antibody levels were quantified using a highly sensitive ELISA method, as outlined previously with specific modifications [22,23]. Initially, 96-well Corning 3369 plates were coated with WG, bovine serum albumin, ovalbumin, peanut, or hazelnut, then subsequently blocked using a 5% gelatin solution. Following a thorough washing step, plasma samples were applied to the plates. Subsequent washes were performed before introducing a biotin-conjugated anti-mouse IgE antibody. After additional washes, streptavidin alkaline phosphatase, and eventually, p-nitro-phenyl phosphate was added to enable quantification, following established methodologies [22,23].

### 4.13. Statistics

Pearson correlation coefficient calculation excel built-in program was used. The following formula was used to calculate *r*-scores:

$$r=\frac{\sum_{i}(x_{i}-\bar{x})(y_{i}-\bar{y})}{\sqrt{\sum_{i}(x_{i}-\bar{x}{)}^{2}}\sqrt{\sum_{i}(y_{i}-\bar{y}{)}^{2}}}$$

Using the *r* scores and *n*-values, significance was calculated with *p* < 0.05. An online software service was used in these analyses (https://www.socscistatistics.com/tests/) (accessed on 1 October 2023). A Student’s *t*-test was used to compare two groups. The statistical significance level was set at *p* < 0.05.

## 5. Conclusions

These data reveal that WG is intrinsically allergenic, and that chronic skin exposure to WG can prime mice for potentially fatal anaphylaxis. Life-threatening anaphylaxis is associated with differential expression of immune biomarkers involved in vascular permeability and allergic immune regulation compared to healthy control mice.

## Figures and Tables

**Table 1:** Identification of immune biomarkers that are significantly increased in the spleen during systemic anaphylaxis.

Biomarker	Control Mice (*n* = 5)	Anaphylactic Mice (*n* = 5)	Student’s *t-*Test *p* <
ACE	38,316.3 ± 1853.51	91,252.75 ± 2471.8	0.001
CD27L	<40 (LOD)	121.18 ± 18.33	0.005
CD30L	7.57 ± 0.44	13.68 ± 0.67	0.001
Dtk	1117.62 ± 26.36	1348.25 ± 46.15	0.005
IL-1a	14.09 ± 2.34	25.88 ± 2.26	0.05
IL-1 R4	391.66 ± 114.88	1022.84 ± 20.13	0.005
IL-2	45.09 ± 1.4	75.5 ± 9.72	0.05
IL-6	59.98 ± 8.13	247.23 ± 26.76	0.001
IL-9	27.2 ± 4.86	124.92 ± 14.87	0.001
IL-12p70	33.78 ± 9.04	78.5 ± 11.13	0.05
IL-15	8185.98 ± 1532.53	25,061.06 ± 3151.03	0.005
IL-17E	44.87 ± 19.9	191.72 ± 89.92	0.05
IL-23	273.89 ± 96.98	939.23 ± 164.11	0.05
MCSF	80.09 ± 2.31	102.92 ± 3.43	0.001
MDC	132.25 ± 5.29	198.63 ± 8.4	0.001
MIG	280.49 ± 12.63	419.55 ± 6.13	0.001
MIP-1a	78.61 ± 7.59	132.23 ± 2.6	0.001
MIP-1g	943.39 ± 9.41	1001.9 ± 10.58	0.01
MIP-2	1.56 ± 0.76	4.65 ± 0.29	0.01
OPG	299.86 ± 5.99	373.37 ± 22.5	0.05
PF4	27,562.44 ± 363.93	31,638.78 ± 629.49	0.001
Prolactin	6.8 ± 1.27	16.57 ± 3.65	0.05
Resistin	152.79 ± 11.04	1237.69 ± 38.04	0.001
SDF-1a	161.28 ± 5.99	325.65 ± 14.87	0.001
VEGF	253.17 ± 6.92	332.56 ± 6.53	0.001
VEGF R3	<20 (LOD)	410.16 ± 105.46	0.01
VEGF-D	1.67 ± 0.57	39.8 ± 3.04	0.001

Statistical significance was determined using Student’s two-tailed test.

**Table 2:** Identification of immune biomarkers that are significantly decreased in the spleen during systemic anaphylaxis.

Biomarker	Control Mice (*n* = 5)	Anaphylactic Mice (*n* = 5)	Student’s *t-*Test *p* <
ALK-1	270.55 ± 10.48	134.18 ± 9.6	0.001
bFGF	3828.16 ± 43.65	3255.51 ± 24.39	0.001
BLC	5639.31 ± 124.83	4678.57 ± 44.06	0.001
CD40	4686.52 ± 208.06	3714.48 ± 242.08	0.05
CD40L	3709.09 ± 69.08	2050.96 ± 74.63	0.001
CTLA4	887.48 ± 10.02	628.25 ± 9.38	0.001
Decorin	14,941.56 ± 302.52	13,997.32 ± 180.47	0.05
Dkk-1	684.12 ± 27.13	521.43 ± 47.91	0.05
Eotaxin	417.45 ± 3.96	184.63 ± 3.05	0.001
Fcg RIIB	6800.55 ± 101.37	5598.51 ± 122.2	0.001
Flt-3L	828.49 ± 8.84	559.04 ± 7.96	0.001
Galectin-1	6404.2 ± 235.85	5393.21 ± 164.45	0.05
Gas 1	1232.06 ± 66.89	974.73 ± 30.9	0.05
GITR	9266.56 ± 301.11	7734.18 ± 336.51	0.05
HGF R	768.41 ± 125.47	394.09 ± 80.25	0.05
IGFBP-2	408.43 ± 23.57	215.84 ± 36.66	0.005
IGFBP-3	2167.55 ± 64.32	1898.07 ± 38.67	0.05
IGFBP-6	1228.83 ± 77.13	541.13 ± 23.11	0.001
IL-1b	14.15 ± 0.7	3.29 ± 0.63	0.001
IL-1ra	618.95 ± 24.81	410.55 ± 14.05	0.001
IL-2 Ra	810.79 ± 36.47	672 ± 26.15	0.05
IL-5	25.43 ± 3.36	<6.8 (LOD)	0.001
IL-12p40	12.32 ± 1.19	3.51 ± 0.91	0.001
IL-17	4.14 ± 0.67	<2.4 (LOD)	0.05
Leptin	911.32 ± 76.19	626.32 ± 34.19	0.05
Leptin R	136.2 ± 28.35	38.75 ± 6.82	0.05
LIX	193.96 ± 2.38	149.09 ± 4.5	0.001
Lymphotactin	9155.09 ± 515.92	3209.5 ± 663.09	0.001
MCP-5	144.37 ± 11.22	86.37 ± 11.55	0.05
MIP-3b	23.78 ± 1.6	2.67 ± 0.78	0.001
OPN	1870.22 ± 44.85	1329.38 ± 67.48	0.001
Pentraxin 3	376.37 ± 66.35	25.64 ± 4.36	0.005
SCF	140.35 ± 3.85	58.14 ± 5.73	0.001
TNF RI	919.47 ± 30.65	720.22 ± 21.12	0.005
TNFα	53.92 ± 7.94	17.62 ± 5.1	0.01
TWEAK R	2839.09 ± 90.38	224.95 ± 82.17	0.001
VEGF R1	436 ± 43.67	269.85 ± 13.02	0.05

Statistical significance was determined using Student’s two-tailed test.

## Data Availability

All the data supporting the results are stored and available at the Institution.

## Supplementary Materials

The following supporting information can be downloaded at: https://www.mdpi.com/article/10.3390/ijms242417247/s1.
