# Supplementary material for: Is Wheat Glutenin Extract Intrinsically Allergenic? Evaluation Using a Novel Adjuvant-Free Mouse Model of Systemic Anaphylaxis
Source: Int J Mol Sci. 2023 Dec 8;24(24):17247. doi: 10.3390/ijms242417247 (PMC10743909; doi:10.3390/ijms242417247)
Supplement: Supplementary file 1 [file ijms-24-17247-s001.zip › ijms-2719244-supplementary.pdf]

A

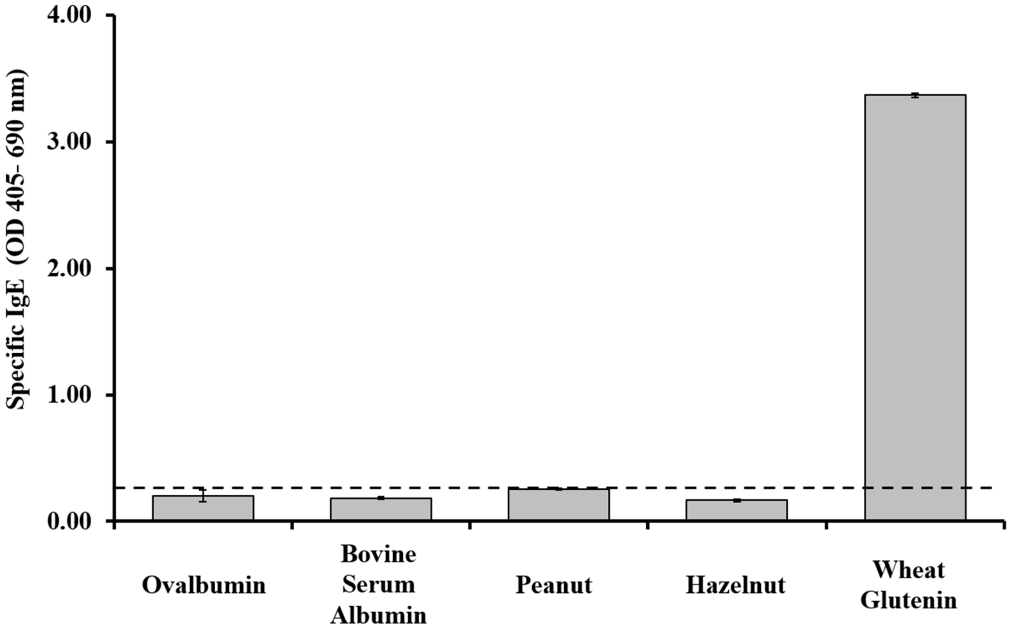

Supplemental Figure S1. IgE antibody elicited by chronic skin exposure to wheat glutenin extract does not bind to other food allergens. Mice were exposed to WG extract as described in Materials and Methods. Blood was collected after 6th exposure. Pooled plasma at 1 in 40 dilution (n=10 sensitized mice) was used in measurement of allergen specific IgE levels (OD 405-690 nm) dilution using an ELISA method described previously. Broken horizontal line represents background activity levels of the assay.

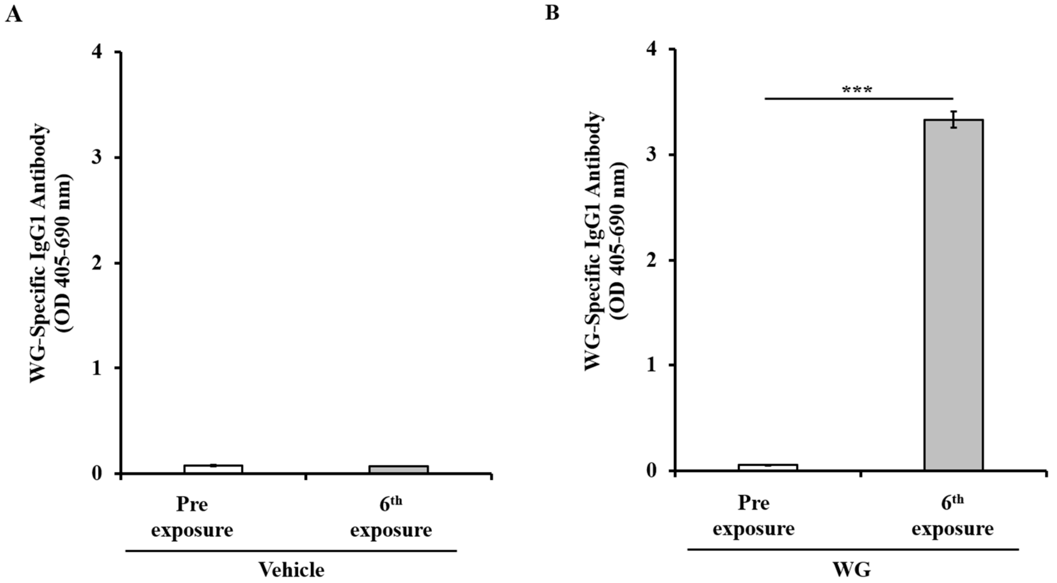

Supplemental Figure S2. Chronic skin exposure to wheat glutenin (WG) elicits IgG1 antibody responses in Balb/c mice. Mice were exposed to WG extract or to vehicle as described in Materials and Methods. Blood was collected before 1st exposure (Pre) and after 6th exposure. Pooled plasma (n = 10 sensitized mice) at 1 in 8000 dilution was used in measurement of IgG1 antibody levels (OD 405-690 nm) using an ELISA method described previously. (A) IgG1 antibody levels in Vehicle-exposed mice. (B) IgG1 antibody levels in WG extract exposed mice. Student's two-tailed t-test: *p < 0.001.
